# Subset Analysis of a Multicenter, Randomized Controlled Trial to Compare Magnifying Chromoendoscopy with Endoscopic Ultrasonography for Stage Diagnosis of Early Stage Colorectal Cancer

**DOI:** 10.1371/journal.pone.0134942

**Published:** 2015-08-13

**Authors:** Tomonori Yamada, Takaya Shimura, Masahide Ebi, Yoshikazu Hirata, Hirotaka Nishiwaki, Takashi Mizushima, Koki Asukai, Shozo Togawa, Satoru Takahashi, Takashi Joh

**Affiliations:** 1 Department of Gastroenterology, Japanese Red Cross Nagoya Daini Hospital, Nagoya, Aichi, Japan; 2 Department of Gastroenterology and Metabolism, Nagoya City University Graduate School of Medical Sciences, Nagoya, Aichi, Japan; 3 Vascular Biology Program and Department of Surgery, Boston Children’s Hospital, Boston, Massachusetts, United States of America; 4 Department of Surgery, Harvard Medical School, Boston, Massachusetts, United States of America; 5 Department of Gastroenterology, Kasugai Municipal Hospital, Kasugai, Aichi, Japan; 6 Department of Gastroenterology, Gifu Prefectural Tajimi Hospital, Tajimi, Gifu, Japan; 7 Department of Gastroenterology, Chukyo Hospital, Nagoya, Aichi, Japan; 8 Department of Experimental Pathology and Tumor Biology, Nagoya City University Graduate School of Medical Sciences, Nagoya, Aichi, Japan; University Hospital Llandough, UNITED KINGDOM

## Abstract

**Background:**

Our recent prospective study found equivalent accuracy of magnifying chromoendoscopy (MC) and endoscopic ultrasonography (EUS) for diagnosing the invasion depth of colorectal cancer (CRC); however, whether these tools show diagnostic differences in categories such as tumor size and morphology remains unclear. Hence, we conducted detailed subset analysis of the prospective data.

**Methods:**

In this multicenter, prospective, comparative trial, a total of 70 patients with early, flat CRC were enrolled from February 2011 to December 2012, and the results of 66 lesions were finally analyzed. Patients were randomly allocated to primary MC followed by EUS or to primary EUS followed by MC. Diagnoses of invasion depth by each tool were divided into intramucosal to slight submucosal invasion (invasion depth <1000 μm) and deep submucosal invasion (invasion depth ≥1000 μm), and then compared with the final pathological diagnosis by an independent pathologist blinded to clinical data. To standardize diagnoses among examiners, this trial was started after achievement of a mean κ value of ≥0.6 which was calculated from the average of κ values between each pair of participating endoscopists.

**Results:**

Both MC and EUS showed similar diagnostic outcomes, with no significant differences in prediction of invasion depth in subset analyses according to tumor size, location, and morphology. Lesions that were consistently diagnosed as Tis/T1-SM_S_ or ≥T1-SM_D_ with both tools revealed accuracy of 76–78%. Accuracy was low in borderline lesions with irregular pit pattern in MC and distorted findings of the third layer in EUS (MC, 58.5%; EUS, 50.0%).

**Conclusions:**

MC and EUS showed the same limited accuracy for predicting invasion depth in all categories of early CRC. Since the irregular pit pattern in MC, distorted findings to the third layer in EUS and inconsistent diagnosis between both tools were associated with low accuracy, further refinements or even novel methods are still needed for such lesions.

**Trial Registration:**

University hospital Medical Information Network Clinical Trials Registry UMIN 000005085

## Introduction

Colorectal cancer (CRC) is the third most common malignancy and the third leading cause of cancer deaths in the world [1]. The 5-year survival rate of CRC is more than 90.3% for localized stage (stage 0, I), 70.4% for regional stage (stage II, III), and 12.5% for distant stage (stage IV) according to the latest statistics from the United States, implying that early diagnosis is critical to curability of this disease [2]. Localized cancer corresponds to mucosal cancer (Tis), T1-, and T2-stage CRC without lymph node metastasis, but Tis/T1-stage CRCs are generally referred to as early stage CRCs in Japan. Among early stage CRCs, mucosal cancer (Tis) and submucosal cancer with slight submucosal invasion (invasion depth <1000 μm, T1-SM_S_) are currently considered an indication for endoscopic resection because of the lack of lymph node metastases, whereas surgical resection with lymph node dissection is recommended for CRC with invasion beyond the deep submucosal layer (invasion depth ≥1000 μm, T1-SM_D_) [3, 4]. Although new endoscopic therapies such as endoscopic submucosal dissection (ESD) enable curative resection for large CRC and pathological diagnosis as a total biopsy [5, 6], ESD for CRC has not yet gained prevalence in Western countries due to its technical difficulty. Accurate pre-diagnosis of the invasion depth of CRC is thus still required to choose the optimal therapy.

Endoscopic ultrasonography (EUS) and magnifying chromoendoscopy (MC) are generally used as diagnostic tools to determine the invasion depth of early CRC, but which modality is better had been unknown. Two prospective studies suggested the advantage of EUS over MC (91.8% *vs*. 63.3%, *P* = 0.0013 [7]; 93% *vs*. 59%, *P*<0.0001 [8]). However, the superiority of EUS remained inconclusive, because the definitions of MC used in those old studies differed slightly from the current definition and the diagnostic order of MC followed by EUS in both studies may have created some biases. On the other hand, recent retrospective studies have shown similar accuracy of determining invasion depth by EUS and MC (75% vs. 87%, *P* = 0.0985 [9]; 82.1% vs. 81.0%, *P* = 0.7785 [10]), but were also inconclusive due to the retrospective designs of the studies. We have recently reported equivalent accuracy between MC and EUS for predicting the invasion depth of early CRC in a multicenter, prospective, comparative study conducted to resolve these questions [11]. However, whether both tools can mutually compensate for information and show some diagnostic differences according to subtype, including tumor size and morphology, remains unclear because there have been no comparative studies between both tools in each category. Such subset analyses provide important information, because we would be able to apply either MC or EUS for a particular type of CRC if either modality proved superior in any category. We therefore conducted subset analyses of the data from our prospective trial to clarify the detailed breakdown of each tool.

## Materials and Methods

### Patients

Inclusion and exclusion criteria for this prospective study have been previously described as follows [11]: 1) adenocarcinoma histologically confirmed by biopsy, including Category 4 or 5 of the Vienna classification [12]; 2) CRC diagnosed as early stage (within submucosal invasion) by conventional endoscopic observation; 3) flat lesions defined (type 0-IIa, slightly elevated; IIb, flat; IIc, slightly depressed); 4) tumor size ≤4 cm; 5) age ≥ 20 years but ≤ 90 years; and 6) Eastern Cooperative Oncology Group performance status (PS) of 0 to 2.

Six Japanese institutions participated in the present trial, recruited patients from February 2011 to December 2012, and the study protocol was approved by the institutional review board (IRB) of the Nagoya City University Hospital (reference number, 46-10-0007), including IRB at other institutions: the IRB of the University of Japanese Red Cross Nagoya Daini Hospital (reference number, 20110322–4), the IRB of Kasugai Municipal Hospital (reference number, 121), the IRB of Gifu Prefectural Tajimi Hospital (reference number, 4), the IRB of Chukyo Hospital (reference number, 2011006), the IRB of Nagoya Memorial Hospital (reference number, 20120125–2). Kasugai Municipal Hospital, Gifu Prefectural Tajimi Hospital and Nagoya Memorial Hospital joined this trial after starting this trial because attending investigators moved to these institutions during this trial. The protocol of this trial and supporting CONSORT checklist are available as supporting information (S1 Protocol, S2 Protocol and S1 CONSORT Checklist).

The trial was carried out according to the ethical guidelines of the 1975 Declaration of Helsinki (6th revision, 2008), and all patients provided written, informed consent before study entry. Before it began, this trial was registered with the University hospital Medical Information Network Clinical Trials Registry (UMIN-CTR) (UMIN000005085).

### Study design

This was a multicenter, randomized, prospective trial that compared the efficacy of MC with EUS in early stage CRC. As shown in the previous primary report [11], patients were randomly assigned to two groups using a computer-aided system at the central research office: Group A, primary MC followed by secondary EUS; and Group B, primary EUS followed by secondary MC. MC and EUS were performed by the same examiner, but the report for the first method was completed before starting the second method and any changes were prohibited for the written reports. The comparison of MC with EUS was performed using all of the data from both groups. To standardize diagnoses among examiners, this trial was started after achievement of a mean κ value of ≥0.6 among all participating endoscopists. To ensure the accuracy and completeness of reporting of studies of diagnostic accuracy, the present randomized controlled trials complied with the STARD initiative [13] and CONSORT guidelines [14].

According to the Japanese Research Society for Cancer of the Colon and Rectum (JSCCR) guidelines [4], depth of vertical submucosal invasion was measured in micrometers from the muscularis mucosae to the deepest cancer ground, in formalin-fixed, paraffin-embedded specimens after resection. This final pathological diagnosis using resected tumor is the gold standard, which was categorized into mucosal invasion <1000 μm (pTis/T1-SM_S_) and deep submucosal invasion ≥1000 μm (≥pT1-SM_D_). In order to calculate accuracy, sensitivity and specificity, the diagnosis before resection by each tool was compared with the final pathological diagnosis of the gold standard. Description of the clinical stages followed the seventh edition of the Union for International Cancer Control tumor-node-metastasis classification [15].

### Diagnostic criteria for the two modalities

The diagnostic definitions for each modality were also previously described, as shown below [11].

#### 1. MC

For MC, all lesions were observed after spraying with 0.05% crystal violet (CV) solution, under 80–100 times imaging using a magnifying colonoscope (CF-H260AZI, PCF-Q240ZI, or CF240ZI; Olympus Optical CO., Tokyo, Japan).

According to Kudo’s pit pattern [16], 5 pit patterns were used, including the following: types I (round pit) and II (asteroid pit), as non-neoplastic patterns; and types III_L_ (regular elongated pit), III_s_ (regular small pit), IV (regular branched pit), V_I_ (irregular pit), and V_N_ (non-structural), as neoplastic patterns including adenoma and cancer. The V_I_ pit pattern with a demarcated area, unclear staining of the area between pits or an unclear outline and irregular margin of the pit was defined as a high-grade irregular V_I_ pit pattern (V_I_-H), and the other V_I_ without these characteristics was defined as low-grade irregular V_I_ (V_I_-L). Type III_L_, III_s_, and IV pit patterns with regular crypts and the V_I_-L pit pattern were clinically defined as an invasion depth of Tis/T1-SM_S_, and type Vi-H and V_N_ pit patterns were defined as ≥T1-SM_D_.

#### 2. EUS

For EUS, all lesions were observed after immersion by distilled water using UM-3R (Olympus Optical CO), which is a 20-MHz, through-the-scope mini probe.

Invasion depth was diagnosed by the findings of the third layer. A hypoechoic area limited to within the first and second layers with the third layer intact and slight irregularity on the surface of the third layer was defined as Tis/T1-SM_S_. A hypoechoic mass that clearly invaded and penetrated into the third layer was defined as ≥T1-SM_D_.

### Sample size and statistical analysis

As described in the primary report of this study [11], the primary end point of this study was diagnostic accuracy for invasion depth. Sixty-two patients for each method were necessary to detect a difference with a two-sided 5% significance level and 80% power using the χ^2^ test, estimating that MC would increase the accuracy for prediction of invasion depth of EUS from 70% to 90%. Finally, the planned sample size was calculated as 70 patients for each method, allowing for about a 10% dropout rate.

The main aim of this subset analysis was to clarify the features of MC and EUC by analyzing diagnostic results from both tools. Data were analyzed using the χ^2^ test or Fisher’s exact probability test, as appropriate, and values of *P* < 0.05 were considered significant. To assess agreement between endoscopists, the Cohen's κ coefficient, which is a measure of agreement beyond chance, was used. This statistic was calculated from the following equation: κ = (Po–Pe)/(1 –Pe), where Po is the proportion of agreement actually observed, and Pe is the proportion of agreement expected by chance. According to the previous proposal, the mean κ value with a 95% confidence interval (CI) for multiple raters was calculated from the average of interobserver agreement between each pair of endoscopists involved in this study [17]. The statistical results were not adjusted for multiple comparison most likely due to the exploratory nature of the trial. Data analyses were performed using Dr. SPSS II for Windows version 11.0.1J software (SPSS Japan, Tokyo, Japan).

## Results

### Patients

**Fig 1:**
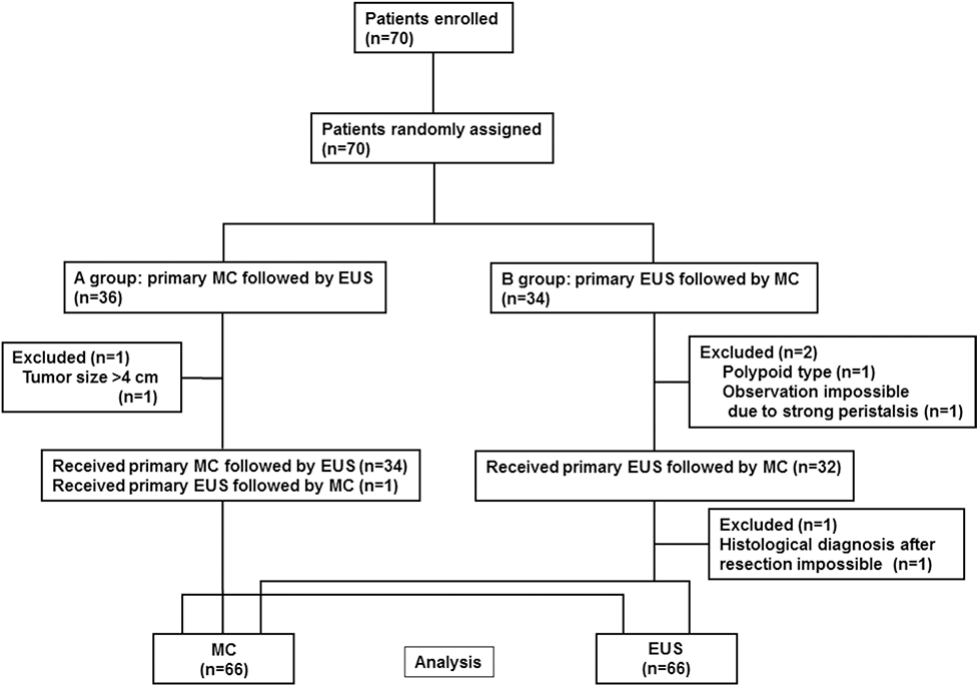
CONSORT flowchart.

Enrollment of this study was closed after collection of scheduled sample size. No adverse events were observed in this study. In total, enrolled 70 patients with 70 lesions were randomly assigned, 36 patients to Group A and 34 patients to Group B. As shown in CONSORT diagram of the previous report (Fig 1) [11], one lesion with tumor size >4cm in A group and one polypoid lesion in B group, which did not fulfill the study criteria, were excluded. One lesion for which observation was impossible due to strong peristalsis and one lesion for which histological diagnosis of invasion depth was impossible due to the severe burning effect of endoscopic resection were also excluded in B group. Finally, the results of MC and EUS were analyzed for a total of 66 lesions.

The patients comprised 49 men and 17 women, with a mean age of 68.7 years. Mean tumor diameter was 19.1 mm, and macroscopic findings were type 0-IIa in 56 lesions and type 0-IIc in 10 lesions. Lesions were located in the rectum in 33 patients and in the non-rectum in 33. Of the 66 patients, 36 underwent endoscopic resection and 30 underwent colectomy with lymph node dissection. The invasion depth of CRC was pTis/T1-SM_S_ in 35 lesions and ≥pT1-SM_D_ in 31 lesions, and all CRCs represented differentiated adenocarcinoma. No significant differences were noted for any factors between Groups A and B. The κ values of MC and EUS reached 0.729 (95%CI, 0.629–0.828) and 0.651 (95%CI, 0.489–0.814) before starting the study.

### Analysis according to tumor size, morphology and location

Among lesions that were consistently diagnosed as Tis/T1-SM_S_ and ≥T1-SM_D_ by both tools, the frequencies of ≥pT1-SM_D_ were 22.2% (6/27) and 76.0% (19/25), respectively. The frequency of ≥pT1-SM_D_ was 40.0% (2/5) among lesions diagnosed as Tis/T1-SM_S_ by MC and ≥T1-SM_D_ by EUS, and 44.4% (5/9) among those diagnosed ≥T1-SM_D_ by MC and Tis/T1-SM_S_ by EUS (Table 1).

**Table 1:** Results according to diagnosis by MC and EUS. Tis/T1-SM_S_, mucosal to submucosal cancer with invasion depth <1000 μm; T1-SM_D_, submucosal cancer with submucosal invasion depth ≥1000 μm.

MC	EUS	≥pT1-SM_D_
Tis/ T1-SM_S_	Tis/ T1-SM_S_	6/27 (22.2%)
Tis/ T1-SM_S_	≥T1-SM_D_	2/5 (40.0%)
≥T1-SM_D_	Tis/ T1-SM_S_	4/9 (44.4%)
≥T1-SM_D_	≥T1-SM_D_	19/25 (76.0%)

Subset analyses according to tumor size, morphology and location in both tools are shown in Table 2. Dividing lesions into ≤20 mm and >20 mm, the accuracy of MC and EUS was similar regardless of size (MC *vs*. EUS: 68.9% *vs*. 72.1%, *P* = 0.818 in ≤20-mm group; 76.2% *vs*. 71.4%, *P* = 0.726 in >20-mm group) (Table 2). Among lesions consistently diagnosed as Tis/T1-SM_S_ by both tools, real frequencies of Tis/T1-SM_S_ in the ≤20-mm and >20-mm groups were 80.0% and 75.0%, respectively. Of the lesions with diagnosis of ≥T1-SM_D_ by both tools, real frequencies of ≥pT1-SM_D_ in the ≤20-mm and >20-mm groups were 73.7% and 83.3%, respectively.

**Table 2:** Subset analysis according to tumor size.

	MC	EUS	≥pT1-SM_D_	*P*
**≤20 mm**	Tis/ T1-SM_S_	Tis/ T1-SM_S_	3/15 (20.0%)	
	Tis/ T1-SM_S_	≥T1-SM_D_	2/5 (40.0%)	
	≥T1-SM_D_	Tis/ T1-SM_S_	2/6 (33.3%)	
	≥T1-SM_D_	≥T1-SM_D_	14/19 (73.7%)	
**Accuracy**	31/45 (68.9%)	32/45 (72.1%)		0.818^#1^
**Sensitivity**	16/21 (76.2%)	16/21 (76.2%)		1.000^#1^
**Specificity**	15/24 (62.5%)	16/24 (66.7%)		0.763^#1^
**>20 mm**	Tis/ T1-SM_S_	Tis/ T1-SM_S_	3/12 (25.0%)	
	Tis/ T1-SM_S_	≥T1-SM_D_	0	
	≥T1-SM_D_	Tis/ T1-SM_S_	2/3 (66.7%)	
	≥T1-SM_D_	≥T1-SM_D_	5/6 (83.3%)	
**Accuracy**	16/21 (76.2%	15/21 (71.4%)		0.726^#1^
**Sensitivity**	7/10 (70.0%)	5/10 (50.0%)		0.650^#2^
**Specificity**	9/11 (81.8%)	10/11 (90.9%)		1.000^#2^

^#1^ χ^2^ test

^#2^ Fisher’s exact probability test. Tis/T1-SM_S_, mucosal to submucosal cancer with invasion depth <1000 μm; T1-SM_D_, submucosal cancer with submucosal invasion depth ≥1000 μm.

The accuracy of MC and EUS was also similar regardless of morphology (MC *vs*. EUS: 73.2% *vs*. 69.6%, *P* = 0.175 in the elevated group; 60.0% *vs*. 80.0%, *P* = 0.628 in the depressed group) (Table 3). Among lesions consistently diagnosed as Tis/T1-SM_S_ by both tools, the real frequency of Tis/T1-SM_S_ in the elevated group was 77.8%. Among lesions with a diagnosis of ≥T1-SM_D_ by both tools, real frequencies of ≥pT1-SM_D_ in the elevated and depressed groups were 76.5% and 75.0%, respectively.

**Table 3:** Subset analysis according to tumor morphology.

	MC	EUS	≥pT1-SM_D_	*P*
**Elevated**	Tis/ T1-SM_S_	Tis/ T1-SM_S_	6/27 (22.2%)	
	Tis/ T1-SM_S_	≥T1-SM_D_	2/5 (40.0%)	
	≥T1-SM_D_	Tis/ T1-SM_S_	4/7 (57.1%)	
	≥T1-SM_D_	≥T1-SM_D_	13/17 (76.5%)	
**Accuracy**	41/56 (73.2%)	39/56 (69.6%)		0.175^#1^
**Sensitivity**	17/25 (68.0%)	15/25 (60.0%)		0.556^#1^
**Specificity**	24/31 (77.4%)	24/31 (77.4%)		1.000^#1^
**Depressed**	Tis/ T1-SM_S_	Tis/ T1-SM_S_	0	
	Tis/ T1-SM_S_	≥T1-SM_D_	0	
	≥T1-SM_D_	Tis/ T1-SM_S_	0/2 (0%)	
	≥T1-SM_D_	≥T1-SM_D_	6/8 (75.0%)	
**Accuracy**	6/10 (60.0%)	8/10 (80.0%)		0.628^#2^
**Sensitivity**	6/6 (100%)	6/6 (100%)		No available
**Specificity**	0/4 (0%)	2/4 (50.0%)		0.429^#2^

^#1^ χ^2^ test

^#2^ Fisher’s exact probability test. Tis/T1-SM_S_, mucosal to submucosal cancer with invasion depth <1000 μm; T1-SM_D_, submucosal cancer with submucosal invasion depth ≥1000 μm.

In addition, the accuracy of MC and EUS was the same in each location (MC *vs*. EUS: 66.7% *vs*. 66.7%, *P* = 1.000 in the rectal group; 75.8% *vs*. 75.8%, *P* = 1.000 in the non-rectal group) (Table 4). Among lesions consistently diagnosed as Tis/T1-SM_S_ by both tools, real frequencies of Tis/T1-SM_S_ in the rectal and non-rectal groups were 78.6% and 76.9%. Among lesions with a diagnosis of ≥T1-SM_D_ by both tools, real frequencies of ≥pT1-SM_D_ in the rectal and non-rectal groups were 61.5% and 91.7%, respectively. Not only accuracy but also sensitivity and specificity between MC and EUC were equivalent regardless tumor size, morphology and location.

**Table 4:** Subset analysis according to tumor location.

	MC	EUS	≥pT1-SM_D_	*P*
**Rectum**	Tis/ T1-SM_S_	Tis/ T1-SM_S_	3/14 (21.4%)	
	Tis/ T1-SM_S_	≥T1-SM_D_	1/2 (50.0%)	
	≥T1-SM_D_	Tis/ T1-SM_S_	2/4 (50.0%)	
	≥T1-SM_D_	≥T1-SM_D_	8/13 (61.5%)	
**Accuracy**	22/33 (66.7%)	22/33 (66.7%)		1.000^#1^
**Sensitivity**	10/14 (71.4%)	9/14 (64.3%)		1.000^#2^
**Specificity**	12/19 (63.2%)	13/19 (68.4%)		0.732^#1^
**Non-rectum**	Tis/ T1-SM_S_	Tis/ T1-SM_S_	3/13 (23.1%)	
	Tis/ T1-SM_S_	≥T1-SM_D_	1/3 (33.3%)	
	≥T1-SM_D_	Tis/ T1-SM_S_	2/5 (40.0%)	
	≥T1-SM_D_	≥T1-SM_D_	11/12 (91.7%)	
**Accuracy**	25/33 (75.8%)	25/33 (75.8%)		1.000^#1^
**Sensitivity**	13/17 (76.5%)	12/17 (70.6%)		1.000^#2^
**Specificity**	12/16 (75.0%)	13/16 (81.3%)		1.000^#2^

^#1^ χ^2^ test

^#2^ Fisher’s exact probability test. Tis/T1-SM_S_, mucosal to submucosal cancer with invasion depth <1000 μm; T1-SM_D_, submucosal cancer with submucosal invasion depth ≥1000 μm.

Assessing lesions with a different diagnosis by MC and EUS, no additional information could not be obtained from either tool in every subset analysis.

### Comparison of detailed findings of MC and EUS with final diagnosis

In terms of the pit pattern classification by MC, accuracies of type III/IV, V_I_-L, V_I_-H and V_N_ were 85.7%, 68.2%, 47.4% and 77.8%, respectively (Table 5). For EUS classification, accuracies of a hypoechoic area within the first-second layers and slight irregularity on the surface of the third layer were 73.3% and 62.5%, whereas accuracies of a hypoechoic area with clear invasion and extensive spread to the third layer were 25.0% and 79.2%, respectively.

**Table 5:** Detailed diagnostic findings of MC and EUS. Tis/T1-SM_S_, mucosal to submucosal cancer with invasion depth <1000 μm; T1-SM_D_, submucosal cancer with submucosal invasion depth **≥**1000 μm.

			Final diagnosis	
MC	Pit pattern type	n	Tis/T1-SM_S_	≥T1-SM_D_
Tis/ T1-SM_S_	III, IV	7	6 (85.7%)	1 (14.3%)
	V_I_-L	22	15 (68.2%)	7 (31.8%)
≥T1-SM_D_	V_I_-H	19	10 (52.6%)	9 (47.4%)
	V_N_	18	4 (22.2%)	14 (77.8%)
**EUS**	**Hypoechoic area**			
Tis/ T1-SM_S_	Within 1^st^-2^nd^ layer	30	22 (73.3%)	8 (26.7%)
	To surface of 3^rd^ layer	8	5 (62.5%)	3 (37.5%)
≥T1-SM_D_	Clear invasion into 3^rd^ layer	4	3 (75.0%)	1 (25.0%)
	Extensive spread to 3^rd^ layer	24	5 (20.8%)	19 (79.2%)

### Difficult lesions for predicting invasion depth

**Fig 2:**
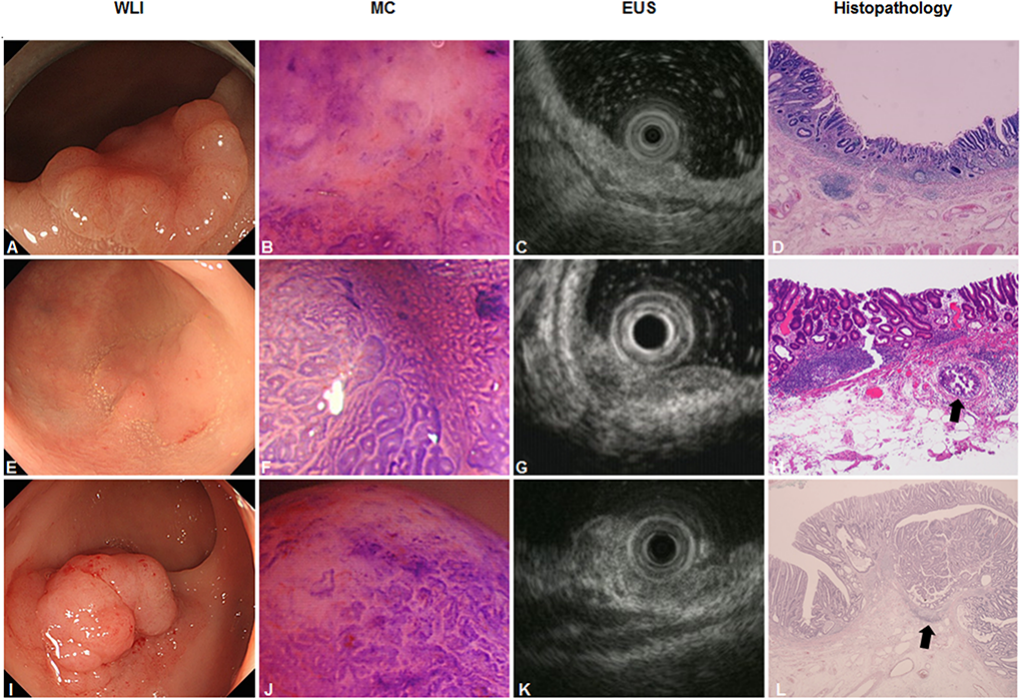
Representative images of difficult lesion for diagnosis. **A-D)** A case of rectal cancer for which diagnosis between magnifying chromoendoscopy (MC) and endoscopic ultrasonography (EUS) was consistent, but incorrect. **A)** Conventional endoscopic view using white-light imaging (WLI) reveals a type 0-IIa+IIc tumor, 10 mm in size. **B)** MC shows a non-structural pit pattern (type V_N_), defined as ≥T1-SM_D_. **C)** EUS shows a hypoechoic mass disrupting the third layer, defined as ≥T1-SM_D_. **D)** Hematoxylin and eosin (HE) staining of the resected specimens shows well-differentiated adenocarcinoma limited to the mucosal layer (pTis) and dense lymphoid follicles in the submucosal layer (×20). No evidence of lymphovascular invasion is seen. **E-H)** A case of sigmoid colon cancer for which diagnosis between MC and EUS was inconsistent, with MC proving correct. **E)** Conventional endoscopic view using WLI reveals type 0-IIa tumor, 20 mm in size. **F)** MC shows a low-grade, irregular, branched pit pattern (type V_I_-L), defined as Tis/T1-SM_S_. **G)** EUS shows a hypoechoic area that clearly invades into the third layer, defined as ≥T1-SM_D_. **H)** HE staining for resected specimens shows well-differentiated tubular adenocarcinoma, mostly limited to mucosal invasion, but submucosal invasive gland (black arrow) was observed with surrounding lymphoid infiltration (×20). The vertical depth of invasion into the submucosa is 250 μm, defined as pT1-SM_S_. There is no evidence of lymphovascular invasion. **I-L)** A case of rectal cancer for which diagnosis between MC and EUS was inconsistent, with EUS proving correct. **I)** Conventional endoscopic view using WLI reveals a type 0-IIa tumor, 20 mm in size. **J)** MC partially shows a non-structural pit pattern (type V_N_) surrounded by a high-grade irregular pit pattern (type V_I_-H), defined as ≥T1-SM_D_. **K)** A hypoechoic area on EUS is confined to the first and second layers, with conservation of the third layer, defined as Tis/T1-SM_S_. **L)** HE staining for resected specimens shows well-differentiated tubular adenocarcinoma, mostly limited to mucosal invasion with submucosal infiltration in a small part (black arrow) (×20). There is no evidence of lymphovascular invasion. The vertical depth of invasion into the submucosa is 500 μm, defined as pT1-SM_S_. There is no evidence of lymphovascular invasion.

Representative images of lesions for which predicting depth of invasion was difficult are shown in Fig 2. Type 0-IIa+IIc rectal cancer of 10mm in diameter was consistently diagnosed as ≥T1-SM_D_ by both MC and EUS (Fig 2A–2C). However, the final histopathological diagnosis was pTis, with dense lymphoid follicles in the submucosal layer (Fig 2D).

Type 0-IIa colon cancer of 20 mm in diameter was inconsistently diagnosed as Tis/T1-SM_S_ by MC and ≥T1-SM_D_ by EUS (Fig 2E–2G), and the final histopathological diagnosis was pT1-SM_s_ (Fig 2H).

Type 0-IIa rectal cancer of 20 mm in diameter was inconsistently diagnosed as ≥T1-SM_D_ by MC and Tis/T1-SM_S_ by EUS (Fig 2I–2K), and the final histopathological diagnosis was pT1-SM_s_ (Fig 2L).

## Discussion

In terms of estimating the depth of invasion in early CRC, our previous report suggested comparable quality between EUS and MC, with 71.2% accuracy [11]. The current updated study analyzed whether EUS or MC offers any superiority according to CRC subtype, including size, morphology and location. Unexpectedly, the diagnostic outcomes for both tools were comparable in every category and no additional information was available from either tool.

Considering artifacts from both tools, attenuation in a large tumor, poor visibility on a fold of the intestinal wall, modification by inside component such as fibrosis and inflammation may decrease accuracy. Whether the diagnostic accuracy of EUS is affected by the size is inconsistent [18, 19], whereas the diagnostic accuracy of MC was not significantly influenced by tumor size in the previous prospective [20] and retrospective studies [21]. In our subset analysis, the diagnostic accuracy was not influenced by the size in both MC and EUS, and no significant differences were found between MC and EUS in ≤20 mm and >20 mm groups.

Although both MC and EUS generally show better accuracy for non-polypoid lesions than for polypoid lesions [21, 22], the diagnostic accuracy of MC was not significantly influenced by morphological classification [20] and some reports for EUS have shown comparable accuracy between elevated and depressed lesions [23]. The influence of morphology in predicting invasion depth by MC and EUS is controversial. Our current results showed similar accuracy regardless of morphology. Since early stage CRCs in our study comprised only flat lesions, the influence of morphology would be low.

As for tumor location, although no studies for MC have been reported, we infer that EUS would be affected by location, because accumulation of water at certain locations can prove difficult for EUS. In fact, a previous study reported that proper scanning by EUS was difficult in 6% (3/49) due to insufficient water in the colonic lumen [24]. However, our current study showed the same accuracy for MC and EUS in both rectum and non-rectum. Observation in the rectum is generally easier than in other areas, but both modalities showed a tendency toward better accuracy for non-rectal CRC than for rectal CRC, implying that tumor location is not associated with successful diagnosis in MC and EUS.

In the present study, even lesions that were consistently diagnosed as Tis/T1-SM_S_ or ≥T1-SM_D_ with both tools revealed accuracy of only 76–78%, suggesting insufficient quality of MC and EUS for evaluating the invasion depth of early CRC. In addition, for CRCs with inconsistent pre-diagnosis between MC and EUS, the actual ratios of pTis/T1-SM_S_ and ≥pT1-SM_D_ were around 60% and 40%, and neither MC nor EUS could provide any additional information in our analysis.

The accuracies of V_I_-L/V_I_-H in MC and surface changes and invasive findings of the 3^rd^ layer in EUS were quite low in the current study (V_I_-L/V_I_-H in MC, 58.5% (24/41); surface change and clear invasion of 3^rd^ layer in EUS, 50.0% (6/12)). Although accuracy of the V_I_-L/V_I_-H pit pattern was reported as 80.8–93.3% / 56.1–78.9% in previous retrospective studies [25, 26] and 93.5% / 87.3% in prospective study [27], distinction between V_I_-L and V_I_-H is sometimes very difficult, with a lack of consistency even between experienced endoscopists [28, 29]. Low accuracy in these diagnostic subsets definitely resulted in poor diagnostic potential in our study. Since the 3^rd^ layer is described as a very thin layer in the colorectal wall, categorizing findings for such a very thin layer would be extremely difficult. A previous study that performed EUS for CRCs with the V_I_ and V_N_ pit pattern, showed 60% accuracy for CRC with V_I_ and 72% accuracy for CRC with V_N_, almost consistent with the current results for MC [23]. Their result also showed that EUS does not provide additional information, consistent with the current study.

The diagnostic difficulty by MC and EUS is affected by not only technical factors but also tumor factors. Inflammatory reaction around tumor results in overstaging for both MC and EUS and it might be sometimes difficult to detect microscopic extension of cancer cells by both tools. In fact, similar cases could be observed in our study: In the first representative case, the overstaging by MC was due to the desmoplastic reaction by inflammation and that by EUS was due to the lymphoid follicles in the submucosal layer; In the second representative case, the overstaging of the EUS would be due to severe lymphoid infiltration in the submucosal layer; In the third representative case, surface mucus attachment may results in overstaging by MC. Novel definition of both tools, methods and technology which can eliminate various bias may be warranted for diagnosis of invasion depth of CRC in the future.

In addition to the two limitations previously described [11], the current subset analysis shows a potential limitation of low statistical power, because each subset comprised only a small sample size. However, use of proper tools depending on the category of CRC is not an essential problem, and analysis of the detailed diagnostic findings for MC and EUS suggested that there is a room for improvement in the diagnostic criteria for each tool.

In conclusion, MC and EUS showed the same diagnostic power for predicting invasion depth in all categories of early CRC and neither study was enough accurate. Since the V_I_ pit pattern in MC, distorted findings to the 3^rd^ layer in EUS and inconsistent diagnosis between both tools were associated with low accuracy, novel diagnostic methods should be developed for such lesions.

## Supporting Information

S1 CONSORT Checklist CONSORT checklist. (DOC)

S1 Protocol Study protcol in Japanese. (PDF)

S2 Protocol Study protocol in English. (PDF)
