# Supplementary material for: Subset Analysis of a Multicenter, Randomized Controlled Trial to Compare Magnifying Chromoendoscopy with Endoscopic Ultrasonography for Stage Diagnosis of Early Stage Colorectal Cancer
Source: PLoS One. 2015 Aug 13;10(8):e0134942. doi: 10.1371/journal.pone.0134942 (PMC4535886; doi:10.1371/journal.pone.0134942)
Supplement: S1 Protocol — (PDF) [file pone.0134942.s002.pdf]

臨床研究課題名

# 「早期大腸癌の深達度診断における拡大内視鏡　対　超音波内視鏡の前向き比較試験」

略語および用語の定義一覧

MC（magnifying colonoscopy）:拡大内視鏡検査

EUS（endoscopic ultrasound）:超音波内視鏡検査

## 0.　臨床試験の概要

### 0-1　目的

早期大腸癌深達度診断における拡大内視鏡検査(MC)と超音波内視鏡検査(EUS)の正診率を前向きに比較検討する。

主要目的：深達度正診率

副次目的：sm 深部浸潤以深癌の感度と特異度、観察時間、A 群の MC と B 群の EUS の深達度正診率、A･B 両群内での深達度正診率

### 0-2　試験デザイン

多施設共同前向き比較試験

### 0-3　試験のフローチャート

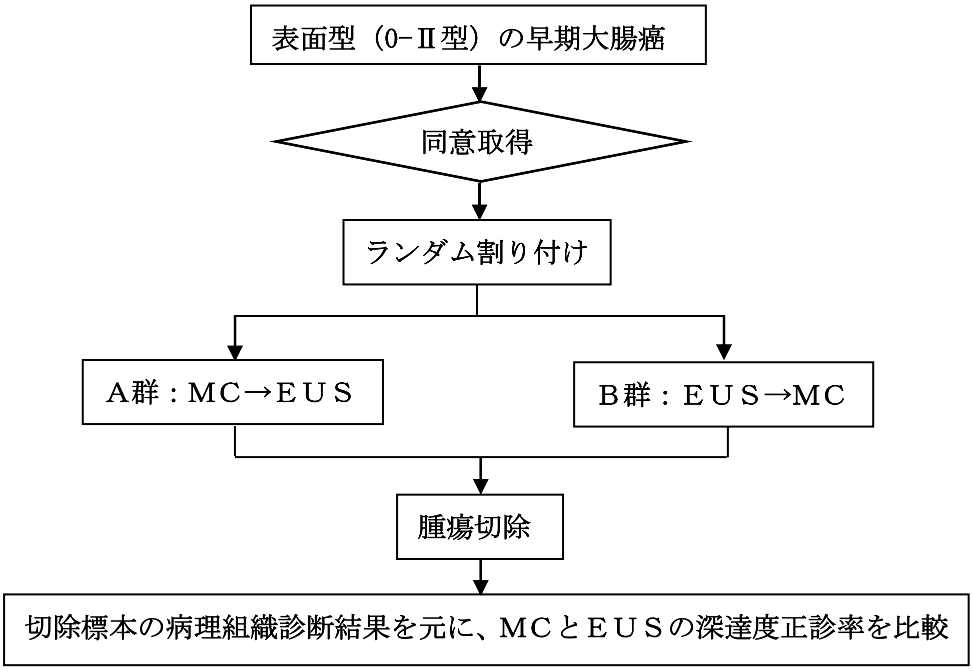


### 0-4　使用可能な医療機器の名称

・拡大内視鏡：　CF-H260AZI または PCF-Q240ZI または CF-Q240ZI

・超音波内視鏡ミニチュアプローブ：UM-3R(20MHz)

### 0-5　試験の対象

＜選択基準＞

① 大腸腫瘍からの生検にて組織学的に腺癌と診断されている(グループ 4 またはグループ 5))。
② 通常の内視鏡観察にて表面型の早期大腸癌と診断される
(大腸癌取扱い規約の肉眼型分類で表面型(0-Ⅱa,Ⅱb,Ⅱc)である(側方進展型腫瘍(LST)も含む))。
③ 腫瘍が 4cm 以下の表面型である。
④ 登録時の年齢が 20 歳以上 90 歳以下である。
⑤ Performance Status(ECOG)が 0,1,2 のいずれかである。
⑥ 試験参加について文書による同意が得られている。

＜除外基準＞

以下のいずれかに該当するものは除外とする。

① 心・脳血管疾患にて強力な抗凝固療法が必要な者。
② 重度に心・肺・腎機能が低下した者
③ 高度な便秘により腸内の洗浄が不可能な者
④ その他、重篤な基礎疾患および全身状態により内視鏡的および外科的切除が困難と判断された者。
⑤ その他医師が不適当と判断した者

## 0-6 試験の方法

**A 群**：最初に腫瘍に色素（0.05%クリスタルバイオレット）を散布し拡大内視鏡観察を行い観察時間を記載するとともに、深達度を診断し EUS を行う前に助手は「症例記録票」に結果を記載する。「症例記録票」への拡大内視鏡所見の記載が完了した後、脱気水を注入し EUS による腫瘍の観察を行い観察時間と診断結果を「症例記録票」に記載する。
**B 群**：A 群とは逆に最初に脱気水を注入後 EUS による腫瘍の観察を行い観察時間と診断結果を「症例記録票」に記載する。「症例記録票」への EUS 診断所見の記載が完了した後、腫瘍に色素（0.05%クリスタルバイオレット）を散布し拡大内視鏡観察を行い観察時間と診断結果を「症例記録票」に記載する。

腫瘍は術前診断や全身状態に応じて通常通り内視鏡的または外科的に切除する。後日切除した腫瘍の固定標本の病理結果と MC および EUS の術前深達度診断の結果を比較検討する。

## 0-7 検査スケジュール

通常大腸内視鏡にて腫瘍を同定後、A 群、B 群ともにその検査順序に従い MC または EUS を続けて一度の内視鏡検査において施行する。

## 0-8 目標症例数

70 例

## 0-9 試験実施期間

2011年2月（IRB承認日）～2013年12月31日

## 1. 試験の背景

早期大腸癌とは粘膜および粘膜下層にとどまる大腸癌のことであり TMN 分類では Tis, T1に分類される。大腸の粘膜内癌(m癌)はほとんどリンパ節転移を来さないと報告されており内視鏡的治療のよい適応とされている(1)。一方、粘膜下層へ浸潤する大腸癌（sm癌）では約13%にリンパ節転移を呈すると報告されておりリンパ節郭清を含んだ腸切除術（外科的治療）が推奨される(2)。しかしながら転移リスクが極めて少ないsm癌が存在しそのような症例に対する腸切除は過剰治療となりうる。近年、本邦における症例集積研究によりsm癌のうち粘膜筋板からの粘膜下層浸潤距離1000μm未満の早期大腸癌はリンパ節転移率が0%であることが報告され(3)、本邦の大腸癌治療ガイドライン2010年版においてはm癌および粘膜下層浸潤距離1000μm未満（sm軽度浸潤癌）の早期大腸癌が内視鏡的切除術の適応とされている(4)。そのため早期大腸癌の病変に応じた最適な治療選択を行うためには、治療前に正確な深達度診断を行うことが重要である。

これら早期大腸癌に対する深達度診断法としては超音波内視鏡（EUS）による腸管壁の断層診断法と拡大内視鏡像（MC）による腺口形態(pit pattern)分類による診断法が有用とされている。

超音波内視鏡診断では、通常大腸癌は低エコー領域として描出され、その腫瘤領域が粘膜下層である第3層まで広がる場合に浸潤距離1000μm以上のsm癌(sm深部浸潤癌)として診断される。過去の報告では超音波内視鏡により 66～88%の深達度正診率が得られると報告されている(5-7)。消化管腫瘍に対するEUS診断のために必要な超音波ミニチュアプローブは安価であり、現在本邦の多くの施設において普及している検査法ではあるが、正確な深達度診断には体位変換を行い病変の存在する腸管に脱気水を貯留させる必要があるためやや煩雑である。

一方拡大内視鏡診断は、腫瘍表面のpit patternを観察することにより腫瘍の良悪性診断および癌の深達度診断を行うものであり拡大内視鏡さえあれば通常の内視鏡観察に引き続いて簡便に検査・診断が可能であることが利点である。しかしながら拡大内視鏡はまだ一部の施設にしか普及していないのが現状である。また各施設間においてpit patternの判定基準、特に深達度診断に重要なV型pit patternの診断基準が微妙に異なっていたことが問題点であったが、2004年の箱根pit patternシンポジウムにてV型pit pattern分類の統一が図られた。腫瘍表面に明らかな無構造領域を有する$V_N$ pit patternおよび既存のpit patternが破壊・荒廃されたVi型高度不整pit patternがsm深部浸潤癌の指標となると定義された。これらの診断基準をもとに拡大内視鏡観察により79～98.8%の深達度正診率が得られると報告されている(8-10)。しかしEUSおよびMCの診断成績はいずれもretrospectiveな検討であるとともに、各々の診断法を専門的に行っている施設からのデータであり、検査法の標準化という意味でのEUSとMCの深達度診断の正確性に関しては不明である。

これまでに早期大腸癌に対するEUSとMCの深達度診断率を比較した前向き試験はふたつ報告されている。Sm深部浸潤の有無の診断率としては、50例の早期大腸癌に対して松本らはEUSがMCに比べ有意に高い正診率を示している（EUS:91.3%、MC:63.3%）(11)。また、Hurlstoneらも52例の早期大腸癌に対してEUSがMCに比べて有意に高い正診率であったと報告している(EUS: 93%、MC: 59%) (12)。しかしながらこれらの試験は、上記の拡大内視鏡診断のV型pit pattern分類の統一以前のデータであり現在の拡大内視鏡pit pattern分類基準においての優劣を示すものとはいえない。ま

た一方、近年報告された内視鏡的切除を施行した早期大腸癌102例におけるMCとEUSの深達度正診率を比較したretrospectie studyでは、EUS: 75%、MC: 83%と両群間に有意差を認めず、むしろ拡大内視鏡検査の正診率が高い傾向にあった(13)。

以上の結果から早期大腸癌における深達度診断としてのEUSとMCの診断成績の優劣に関しては議論の余地があり不明である。現状では大腸癌術前深達度診断法の標準的診断法は確立されておらず、施行検査法は施設の機材状況や術者に依存している状況である。前述のごとくEUSに比べてMCは短時間で簡便に施行可能である可能性があるとともに、MCのEUSに対する優越性が証明されれば早期大腸癌に対する標準的診断法としての位置づけが確立されると考えられ本試験を計画した。

## 2. 試験の目的

早期大腸癌に対する超音波内視鏡と拡大内視鏡での深達度診断能の有効性を手術後の病理組織と前向きに比較検討する。

主要目的：深達度正診率(m癌・sm軽度浸潤癌およびsm深部浸潤癌)

副次目的：sm深部浸潤以深癌の感度と特異度、観察時間、A群のMCとB群のEUSの深達度正診率、A･B両群内での深達度正診率

## 3. 使用可能な医療機器の情報

- 拡大内視鏡： CF-H260AZIまたはPCF-Q240ZIまたはCF-Q240ZI
- 超音波内視鏡ミニチュアプローブ：UM-3R(20MHz)

## 4. 試験の対象

### 4-1 選択基準

① 大腸腫瘍からの生検にて組織学的に腺癌と診断されている(グループ4またはグループ5)。
グループ4：腫瘍性で高度異型を示す病変。
グループ5：明らかに癌と診断しうる異型を示す病変。

② 通常の内視鏡観察にて表面型の早期大腸癌と診断される
(肉眼型分類で表面型(0-Ⅱa,Ⅱb,Ⅱc)である(側方進展型腫瘍(LST)も含む))。
0-Ⅱa:表面隆起型, 0-Ⅱb:表面平坦型, 0-Ⅱc:表面陥凹型

③ 腫瘍が4cm以下である。

④ 登録時の年齢が20歳以上90歳以下である。

⑤ Performance Status(ECOG)が0,1,2のいずれかである。

⑥ 試験参加について文書による同意が得られている。

### 4-2 除外基準

以下のいずれかに該当するものは除外とする。

① 心・脳血管疾患にて強力な抗凝固療法が必要な者。

② 重度に心・肺・腎機能が低下した者。

③ 高度な便秘(摘便を要する頑固な便秘)により腸内の洗浄が不可能な者。

④ その他、重篤な基礎疾患および全身状態により内視鏡的および外科的切除が困難と判断した者。

⑤ その他医師が不適当と判断した者

## 5. 試験デザイン

多施設共同前向き比較試験

## 6. 試験への登録方法とその手続き

### 6.1. 術者間の診断基準の統一

試験開始前に撮影画像を用いてMCおよびEUSの深達度診断の講習訓練を行いκ値を算出し、試験担当医師間において7.2.2.の診断基準の統一を計る。

### 6.2. ランダム割付

登録にあたって検査群への割り付けを登録順に単純ランダム化法により行う。

試験開始前に乱数に基づき割付表を作成し臨床試験現場とは独立した部署(名古屋市立大学消化器代謝内科学　秘書室)にてランダムに割り付けられる。

### 6.3. 登録の手順

1) 各施設の臨床試験審査委員会（IRB）承認書の「写し」を郵送又はFAX送信、PDF化文書の送信等にて研究事務局へ提出後に開始することができる。
2) 各施設の試験責任医師及び試験分担医師は患者（被験者候補）への説明を開始し、試験責任医師はスクリーニング名簿に患者氏名等を記載する。
3) 各施設の試験責任医師は、本試験への同意の有無をスクリーニング名簿に記載し、同意取得順に各施設毎に被験者識別コード2桁（01～）を付番する。
4) 本試験に同意を得た後、試験担当医師は、「登録票」に必要事項を記入し、研究事務局にFAX送信する。
5) 「登録票」を受信した研究事務局は、「登録通知票」に登録番号および試験群を記入して各施設にFAXを返信する。
6) 実施にあたり、試験担当医師は「症例記録票」に記入する。
7) 各施設の試験責任医師は、「スクリーニング名簿」、「登録票/登録通知票」、「症例記録票」を管理保管する。
8) 定められた検査は、登録後速やかに開始する。

## 7. 試験方法

### 7.1. プロトコール診断法

通常の大腸内視鏡検査に引き続き下記のプトコール診断法に基づき深達度診断を行う。

#### 7.1.1. A群：MC→EUS

① 腫瘍に色素(0.05%クリスタルバイオレット)を散布し拡大内視鏡観察を行い、診断の根拠となる写真を撮影する。
② 色素散布開始時から拡大内視鏡観察終了までの時間（分・秒）をストップウォッチで測定し拡大内視鏡診断の観察時間として記載する。
③ 超音波内視鏡検査施行前に助手は「症例記録票」に拡大内視鏡検査の深達度診断結果を記載する。
④ 色素を洗浄吸引後、脱気水を注入し超音波内視鏡診断を行い、診断の根拠となる写真を撮影する。
⑤ 脱気水注入開始時から超音波内視鏡診断終了までの時間（分・秒）をストッ

プウォッチで測定し超音波内視鏡診断の観察時間として記載する。

⑥ 助手は「症例記録票」に超音波内視鏡検査の深達度診断結果を記載する。

7.1.2. B群：EUS→MC

① 脱気水を注入し超音波内視鏡診断を行い、診断の根拠となる写真を撮影する。

② 脱気水注入開始時から超音波内視鏡診断終了までの時間（分・秒）をストップウォッチで測定し超音波内視鏡診断の観察時間として記載する。

③ 深達度を診断し、助手は「症例記録票」に超音波内視鏡検査の深達度診断結果を記載する。

④ 脱気水を吸引後、腫瘍に色素(0.05%クリスタルバイオレット)を散布し拡大内視鏡観察を行い、診断の根拠となる写真を撮影する。

⑤ 色素散布開始時から拡大内視鏡観察終了までの時間（分・秒）をストップウォッチで測定し拡大内視鏡診断の観察時間として記載する。

⑥ 助手は「症例記録票」に拡大内視鏡検査の深達度診断結果を記載する。

7.1.3. 検査終了後腫瘍切除

① 試験担当医師は、検査後7日以内に「症例記録票」を研究事務局に提出(FAX も可）する。

② 術前の深達度診断結果や全身状態に応じて腫瘍は内視鏡的または外科的に切除する。

7.1.4. 病理組織検体の提出

① 試験担当医師は各症例の「治療方法/病理診断票」とともに大腸癌の切除標本の薄切プレパラートを各切片3 枚ずつ添えて研究事務局へ郵送する。

② (送付先)

〒467-8601
名古屋市瑞穂区瑞穂町字川澄1番地
名古屋市立大学大学院医学研究科　消化器代謝内科学
志村　貴也、　海老　正秀

③ 切除術後の大腸癌組織標本を用いて、大腸癌の深達度および脈管侵襲の有無、簇出の有無について術前の深達度診断結果を盲検化された独立した病理診断医により病理診断を行い、その結果を「症例記録票」に記載する。

7.1.5. 病理組織検体の保存

病理組織プレパラートの保管場所は名古屋市立大学大学院医学研究科消化器代謝内科学の研究室とする。

7.2. 深達度診断の分類と定義

7.2.1. 深達度診断の分類

早期大腸癌の深達度診断は、内視鏡的切除か外科的切除かの治療選択法に基づき、「①m 癌または sm 軽度浸潤癌」、「②sm 深部以深浸潤癌」の 2 つに分類する。

7.2.2. 深達度診断の定義

**① 拡大内視鏡診断**

**m 癌/sm 軽度浸潤癌：** IIIs, $III_L$, IV, $V_i$ 軽度不整 pit pattern

規則正しい pit または 領域を持たない不整 pit。

**sm 深部以深浸潤癌**：$V_i$ 高度不整 pit pattern(領域をもって既存の pit pattern が破壊、荒廃したもの)。

$V_N$ 型 pit pattern(明らかな無構造領域を有するもの)。

② **超音波内視鏡診断**

**m 癌/sm 軽度浸潤癌**：腫瘍が第 1～2 層に限局するもの/腫瘍により第 3 層上縁が軽度の不整・狭小化を認めるもの。

**sm 深部以深浸潤癌**：腫瘍により第 3 層が明らかに狭小化・断裂をきたすもの。

## 8. 検査・観察項目および検査実施の時期

| 観察項目 | 同意取得前 | 同意取得時 | 大腸内視鏡下試験時 | 大腸内視鏡下試験 30 分後 |
|---|---|---|---|---|
| 患者背景 | ● | | | |
| 大腸内視鏡検査 | ● | | | |
| 同意取得 | | ● | | |
| 登録 | | ● | | |
| 診察（腹痛・下血の有無） | | ● | | ● |
| 酸素飽和度 | | | ● | |
| 血圧測定 | | | ● | ● |
| EUS と MC の癌深達度所見 | | | ● | |
| EUS と MC の観察時間測定 | | | ● | |

## 9. 併用禁止薬および併用禁止療法

特に設定しない。

## 10. 被験者の中止基準

以下の場合は、本試験を中止し適切な措置を講ずる。

1) 患者から試験中止の申し出があった場合。
2) 試験が困難な有害事象が認められた場合。
3) その他、試験責任医師または試験分担医師が困難と判断した場合。

## 11. 試験の評価項目

### 11-1 主要評価項目

**深達度正診率**：術前に施行したMCとEUSの深達度診断結果と治療後の最終病理組織検査結果とを対比し、術前に「m癌・sm軽度浸潤癌」または「sm深部以深浸潤癌」と診断が一致した腫瘍の割合。

### 11-2 副次的評価項目

**sm 深部浸潤以深癌の感度と特異度**：術後の病理組織結果で sm 深部以深浸潤癌と診断された腫瘍を術前に sm 深部以深浸潤癌と正しく診断した確率(感度)。術後の病

理組織結果でm癌またはsm軽度浸潤癌と診断された腫瘍を術前にm癌またはsm軽度浸潤癌と正しく診断した確率（特異度)。

**観察時間**： MCおよびEUSの各々の診断法の検査開始から終了までの時間。

- **MC**：色素(クリスタルバイオレット)散布開始時から拡大内視鏡観察終了までの時間(分・秒)。
- **EUS**：脱気水注入開始時から超音波内視鏡観察終了までの時間(分・秒)。

**A群のMCとB群のEUSの深達度正診率**：A群のMCとB群のEUS(つまり各群の最初に行った検査法)により、術前に「m癌・sm軽度浸潤癌」または「sm深部以深浸潤癌」と正しく診断された腫瘍の割合。

**A･B 両群内での深達度正診率**：A 群・B 群各群内での MC と EUS による深達度正診率。

## 12. 有害事象の報告および被験者の安全性確保

### 12-1　有害事象の定義

有害事象とは、試験に際して観察される、あらゆる好ましくない意図しない徴候（臨床検査値の異常も含む)・症状・疾患であり、試験治療との因果関係は問わない。すなわち因果関係があると判断されるものと、因果関係ありと判断されないもの両者を含む.

### 12-2　有害事象の評価

- 有害事象の評価規準（Toxicity grading criteria）として以下①を用いる。
  - ①　有害事象共通用語規準v4.0 日本語訳JCOG/JSCO 版（NCI Common Terminology Criteria for  Adverse Events v4.0（CTCAE v4.0））

- 有害事象が認められた場合、grading しカルテに記録を残す。

### 12-3　重篤な有害事象の評価と報告

有害事象の「重篤」とは、治療された際に生じたあらゆる好ましくない医療上の出来事のうち、以下のものに規定した。

①　死に至るもの
②　生命を脅かすもの（発現時点において患者が死の危険にさらされている場合)
③　治療のための入院または入院期間の延長が必要となるもの
④　永続的または顕著な障害・機能不全に陥るもの
⑤　その他の医学的に重要な状態と判断される事象または反応

- 報告すべき期間は試験終了後 30 分以内とする。ただし、規定された有害事象情報の収集期間が終了した後であっても、試験との因果関係が疑われる重篤な有害事象については同様に報告する。
- 各施設の試験責任医師は、重篤な有害事象の内容を「医療機器安全性情報報告書」に記載し、研究事務局へ FAX 送信する。

・各施設の試験責任医師は、試験との因果関係の有無にかかわらず、「重篤な有害事象」を各施設の有害事象の報告手順に従って施設長へ速やかに報告する。

### 12-4　予期される有害事象

通常の大腸内視鏡検査と同様に起こりうる有害事象として下記の事象が上げられる。

大腸穿孔、消化管出血、腸閉塞、血栓症/塞栓症、心臓-虚血/梗塞、上室性不整脈、心室性不整脈、中枢神経系脳血管系虚血、発熱、低体温、血圧低下、アレルギー反応/過敏症、

## 13. 試験全体の中止基準

### 13-1 試験の終了

試験責任医師は、予定症例数に対して試験実施計画書に規定された観察及び検査が終了後、各施設内の取り決めに従い、終了の手続きを行う。

### 13-2 試験の中止又は中断

研究代表者は安全性あるいは有効性の見地から、試験を中止する必要が生じた場合、速やかにその旨及び理由を各施設の試験責任医師へ文書で報告する。

## 14. データの取り扱い・公表に関する取り決め

臨床試験審査委員会の承認が得られた後、大学病院医療情報ネットワーク(UMIN)「臨床試験登録システム」へ登録する。

試験責任医師及び試験分担医師は、本試験により得られた結果の一部又は全部を結果の如何に関わらず学会、学術雑誌等に公表する。

## 15. 試験実施期間

2011年2月（IRB承認日）～2013年12月31日

## 16. 統計学的事項

### 16-1 主たる解析と判断規準

主要評価項目である深達度正診率について、カイ二乗検定を用いて検討を行い有意水準は両側5%とする。

### 16-2 目標症例数および設定の根拠

対照検査群である超音波内視鏡診断法に対して、試験検査群の拡大内視鏡検査法が深達度正診率において優れていることを検証する。

EUSの深達度正診率が70%と仮定し、MCにより90%への正診率の増加が見込めることを検証する。αエラー=0.05、検出力：80%とした場合必要症例数は各群62例となる。1例に対してMCおよびEUSの両方の検査を可能であることから必要症例数は62例となる。

同意の取り消しや前処置不良による深達度判定不能など何らかの原因で約1割の症例が脱落すること考慮し本試験の必要症例数は70例と設定した。

### 16-3 最終解析

主要評価項目である深達度正診率を算出する。

## 17. 倫理的事項

### 17-1 実施計画書の遵守

試験計画書を遵守して実施する。

### 17-2 遵守すべき諸規則（被験者の保護）

「ヘルシンキ宣言」および「臨床研究に関わる倫理指針の精神」に基づき、患者の人権、安全及び福祉に対して配慮するとともに、患者のプライバシーを尊重し、機密保持に努める。症例報告書の作成、原医療記録等の直接閲覧・取扱いにおいて、登録番号で特定する等、患者の機密保持に努める。

### 17-3 同意の方法およびインフォームド・コンセントの手順

各施設の臨床試験審査委員会で承認された説明・同意文書を十分に説明し、説明医師署名欄に説明日および署名を記載する。この臨床試験への参加について、ご本人の自由意思による同意を文書により取得する。

名古屋市立大学病院においては説明・同意文書を 3 部準備し、試験責任医師及び試験分担医師は、3 部の説明・同意文書の署名欄の日付およびご本人の署名を確認後、一部はご本人に交付、一部は診療録に保管（病歴センターにてスキャンして取り込み指示）し、残りの一部を臨床試験管理センターにて保管する。

### 17-4 説明同意文書の項目と改訂

1. 臨床試験について
2. 臨床試験の目的
3. 臨床試験の責任医師の氏名及び分担医師の氏名
4. 臨床試験の方法及び期間
5. 期待される利益と予測される不利益
6. 他の診断方法の有無及びその内容
7. この臨床試験に参加しなくても不利益を受けることはないこと
8. あなたのプライバシーに係わる内容は保護されること
9. 健康被害の補償に関すること
10. 臨床試験についてさらに詳しい情報が欲しい場合および健康状態に問題があると感じられた時の医療機関の連絡先
11. この臨床試験に参加する場合の費用について
12. その他(新たな情報、中止する場合があること)

被験者の意思に影響を与える可能性のある新たな重要な情報（通常、説明・同意文書の改訂を必要とする情報）が得られた場合には、試験責任医師は速やかに当該情報に基づき説明・同意文書を改訂し、あらかじめ臨床試験審査委員会の承認を得る。

### 17-5 個人情報の保護

個人を特定できるような情報を保護する。

## 18. 健康被害の補償

試験により何らかの健康被害が生じた際、病院は必要な治療を保険診療で提供し、特別な補償金が支払われることはない。

## 19. 試験の費用負担

### 19-1 本試験に伴い発生する利益相反

本試験において利益相反行為はない

### 19-2 試験治療に関する費用

「臨床試験に係る費用」は、保険診療の範囲で被験者が全額負担する。

大腸癌切除標本のプレパラートの送付にかかる費用は各施設の負担とする。

## 20. 実施計画書の改訂

研究代表者が実施計画書の改訂が必要と考えた場合に、臨床試験審査委員会の承認を得て行なう。

## 21. 研究組織

### 21-1 研究代表者

名古屋市立大学大学院医学研究科
試験責任医師　消化器・代謝内科学　志村貴也
〒467-8601　名古屋市瑞穂区瑞穂町川澄 1
連絡先：TEL　052-853-8211　FAX　052-852-0952

### 21-2 研究事務局

名古屋市立大学大学院医学研究科　消化器・代謝内科学
〒467-8601　名古屋市瑞穂区瑞穂町川澄 1
連絡先：TEL　052-853-8211　FAX　052-852-0952

### 21-3 実施計画書作成者

名古屋市立大学大学院医学研究科　消化器・代謝内科学
志村貴也

### 21-4 病理診断医

名古屋市立大学病院　病理検査部
高橋　智

## 22. 研究参加施設および責任医師

| | |
|---|---|
| 名古屋市立大学病院 | 海老正秀 |
| 名古屋第二赤十字病院 | 山田智則 |
| 社会保険中京病院 | 戸川昭三 |

## 23. 引用文献

1. Fujimori T, Kawamata H, Kashida H. Precancerous lesions of the colorectum. J Gastroenterol 2001; 36: 587-94.

2. Nascimbeni R, Burgart LJ, Nivatvongs S, Larson DR. Risk of lymph node metastasis in T1 carcinoma of the colon and rectum. Dis Colon Rectum 2002; 45: 200-6.
3. Kitajima K, Fujimori T, Fujii S, et al. Correlations between lymph node metastasis and depth of submucosal invasion in submucosal invasive colorectal carcinoma: a Japanese collaborative study. J Gastroenterol 2004; 39: 534-43.
4. 大腸癌研究会編. 大腸癌治療ガイドライン-医師用 2010 年版. 金原出版 2010; 東京.
5. Konishi K, Akita Y, Kaneko K, et al. Evaluation of endoscopic ultrasonography in colorectal villous lesions. Int J Colorectal Dis 2003; 18: 19-24.
6. Frascio F, Giacosa A. Role of endoscopy in staging colorectal cancer. Semin Surg Oncol 2001; 20: 82-5.
7. 小林清典, 木田光広, 勝又伴栄, et al. 早期大腸癌の深達度診断および治療法選択における超音波内視鏡診断の臨床的意義. Gastroenterol Endoscopy 2004; 46: 2132-40.
8. Kato S, Fujii T, Koba I, et al. Assessment of colorectal lesions using magnifying colonoscopy and mucosal dye spraying: can significant lesions be distinguished? Endoscopy 2001; 33: 306-10.
9. Matsuda T, Fujii T, Saito Y, et al. Efficacy of the invasive/non-invasive pattern by magnifying chromoendoscopy to estimate the depth of invasion of early colorectal neoplasms. Am J Gastroenterol 2008; 103: 2700-6.
10. Bianco MA, Rotondano G, Marmo R, et al. Predictive value of magnification chromoendoscopy for diagnosing invasive neoplasia in nonpolypoid colorectal lesions and stratifying patients for endoscopic resection or surgery. Endoscopy 2006; 38: 470-6.
11. Matsumoto T, Hizawa K, Esaki M, et al. Comparison of EUS and magnifying colonoscopy for assessment of small colorectal cancers. Gastrointest Endosc 2002; 56: 354-60.
12. Hurlstone DP, Brown S, Cross SS, Shorthouse AJ, Sanders DS. High magnification chromoscopic colonoscopy or high frequency 20 MHz mini probe endoscopic ultrasound staging for early colorectal neoplasia: a comparative prospective analysis. Gut 2005; 54: 1585-9.
13. Fu KI, Kato S, Sano Y, et al. Staging of early colorectal cancers: magnifying colonoscopy versus endoscopic ultrasonography for estimation of depth of invasion. Dig Dis Sci 2008; 53: 1886-92.
