# Supplementary material for: Subset Analysis of a Multicenter, Randomized Controlled Trial to Compare Magnifying Chromoendoscopy with Endoscopic Ultrasonography for Stage Diagnosis of Early Stage Colorectal Cancer
Source: PLoS One. 2015 Aug 13;10(8):e0134942. doi: 10.1371/journal.pone.0134942 (PMC4535886; doi:10.1371/journal.pone.0134942)
Supplement: S2 Protocol — (PDF) [file pone.0134942.s003.pdf]

**Title of the study**

**"Magnifying chromoscopic colonoscopy versus endoscopic ultrasonography to predict depth of invasion for early colorectal cancer"**

# 0. Summary of the study

## 0-1 Study aim

To prospectively compare magnifying chromoendoscopy (MC) with endoscopic ultrasonography (EUS) for preoperative diagnosis of invasion depth in early colorectal cancer (CRC)

Primary endpoint: accuracy of invasion depth
Secondary endpoint: Sensitivity and specificity for deep submucosal invasion, Observation time, Accuracy rate between MC in A group and EUS in B group, Accuracy rate in each group between MC and EUS

## 0-2 Study design

Multicenter, prospective comparative trial

## 0-3 Study flowchart

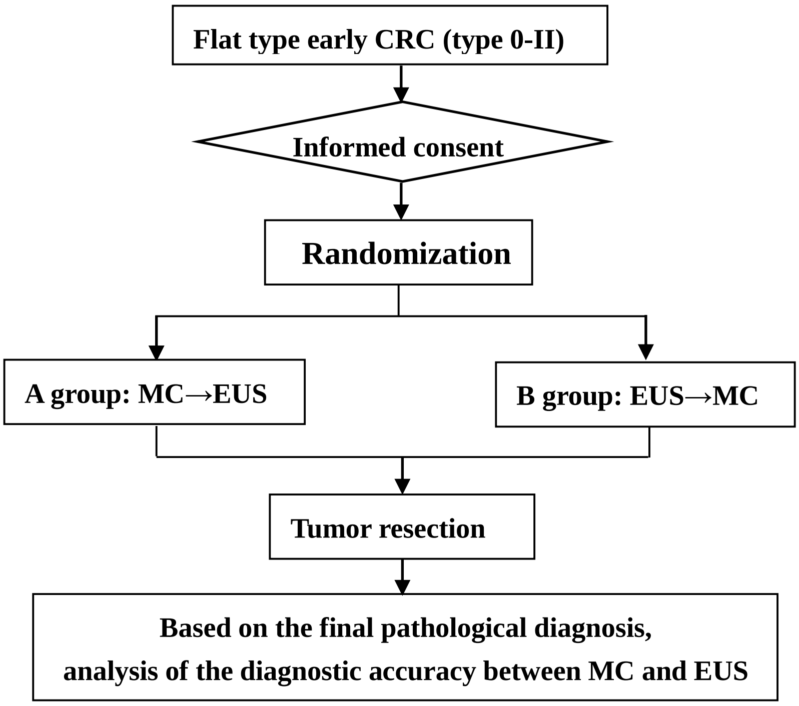


## 0-4 Endoscopy and EUS

- MC: CF-H260AZI, PCF-Q240ZI or CF-Q240ZI
- EUS: Through-the-scope mini probe, UM-3R(20MHz)

**0-5 Objectives**

**(Inclusion criteria)**

1. Histologically confirmed adenocarcinoma by biopsy, including Category 4 or 5
2. CRC diagnosed as flat-type and early stage by conventional endoscopic observation
3. Tumor size ≤4 cm
4. 20 ≤ age ≤ 90 years
5. Eastern Cooperative Oncology Group performance status (PS) of 0 to 2
6. Completion of informed consent (IC) to this trial

**(Exclusion criteria)**

1. Patients with necessity to heparinize during endoscopy
2. Patients with severe heart, lung and renal dysfunction
3. Patients with severe constipation
4. Debilitated patients who cannot undergo endoscopic or surgical resection
5. Inappropriate patients by each investigator’s judgement

**0-6 Methods**

**A group**: First, all lesions are observed after spraying with 0.05% crystal violet, under 80-100 times imaging using a magnifying colonoscopy. An assistant writes observation time and the diagnosis of invasion depth by primary MC in the case report form (CRF). After that, distilled water is injected into colon/rectum through endoscopy and the investigator can start observation by secondary EUS. Likewise, the report of secondary EUS are completed.

**B group:** In contrast to the A group, first, distilled water is injected into colon/rectum through endoscopy and the investigator start EUS. An assistant writes observation time and the diagnosis of invasion depth by primary EUS in the CRF. After that, the investigator can start observation by secondary MC after spraying with 0.05% crystal violet.
Likewise, the report of secondary EUS are completed.

Each tumor is appropriately resected with endoscopy or surgery and pre-diagnosis of invasion depth by MC and EUS are collated to the final pathological results.

**0-7 Schedule of the examination**

The endoscopist sequentially performs MC and EUS based on the rule of either A or B group, after detection the tumor by standard endoscopic observation.

**0-8 Sample size**

70 cases

**0-9 Trial term**

February, 2011 – December 31, 2013

## 1. Background

Early stage CRC is colorectal caner within mucosal and submucosal invasion that is categorized into stage Tis and T1 according to theTNM classification. Intramucosal CRC (M) is a good indication for endoscopic resection because there is no probability of lymph node metastasis (LNM) (1). On the other hand, surgical resection is generally recommended for submucosal CRC because of about a 13% possibility of LNM (2). However, surgical resection for all submucosal CRCs would result in over-surgery because there are some submucosal CRCs with quite low risk of LNM.

As a recent Japanese study has reported no LNM in submucosal cancer with slight submucosal invasion (invasion depth <1000 µm, $SM_S$) (3), M/SMs has been currently considered an indication for endoscopic resection (4).

Therefore, diagnosis of invasion depth before treatment is the most important for choosing the therapeutic strategy of early CRC. Both EUS and MC are considered as useful methods for the pre-diagnosis of invasion depth of early CRC. Tumor can be generally described as low echoic lesion by EUS and its spread to the 3rd layer are diagnosed as submucosal CRC with deep submucosal invasion (invasion depth ≥1000 µm, $SM_D$). Previous reports have shown 66-88% accuracy with EUS for diagnosis of invasion depth for early CRC (5-7). Although EUS for early CRC has been widely spread in Japanese clinical setting, performance of EUS is somewhat complicated because EUS requires distilled water injection and changing body position during procedure.

Meanwhile, MC enables diagnosis of invasion depth of CRC by morphological evaluation of surface crypts on the tumor, which is called the pit pattern. MC can be quickly performed after standard endoscopic observation. The pit pattern is roughly divided into 5 patterns, and type V, which shows a fairly irregular and non-structural pit pattern, is a match for cancer. The classification of type V pit pattern was complex, but type V pit patterns with severe irregularity and a non-structural appearance are reported as cancer with $SM_D$ at the consensus symposium in 2004. Based on these diagnostic criteria, MC has reportedly shown 79-98.8% accuracy for diagnosis of invasion depth for early CRC (8-10). However, real efficacy of each tool remained unclear because these results of EUS and MC were reported as single arm study from special institution for each method.

There have been two prospective studies to compare MC with EUS for the prediction of invasion depth of CRC so far. Both studies showed that EUS was consistently superior to MC with a higher accuracy for diagnosis of invasion depth (91.8% *vs.* 63.3%, $P$=0.0013 (11); 93% *vs.* 53%, $P$<0.0001 (12)). However, results of these trials were based on the previous definition before standardization of MC pit pattern, and the comparison of EUS and MC under the current pit pattern classification have been unknown. The latest retrospective study showed a better tendency of MC compared to EUS (MC: EUS, 87% vs. 75%, $P$=0.0985) although it was not significant (13).

From these previous results, standard diagnostic method for invasion depth of early CRC is still inconclusive and its usage depends on a choice of each institution and endoscopist. We thus planed this trial to prove the speriority of MC to EUS.

## 2. Study aim

To prospectively compare MC with EUS for preoperative diagnosis of invasion depth in early colorectal cancer (CRC)

Primary endpoint: accuracy of invasion depth

Secondary endpoint: Sensitivity and specificity for deep submucosal invasion, Observation time, Accuracy rate between MC in A group and EUS in B group, Accuracy rate in each group between MC and EUS

## 3. Endoscopy and EUS

- MC: CF-H260AZI, PCF-Q240ZI or CF-Q240ZI
- EUS: Through-the-scope mini probe, UM-3R (20MHz)

## 4. Objectives

### 4-1 Inclusion criteria

1. Histologically confirmed adenocarcinoma by biopsy, including Category 4 or 5
2. CRC diagnosed as flat-type and early stage by conventional endoscopic observation
3. Tumor size ≤4 cm
4. 20 ≤ age ≤ 90 years
5. Eastern Cooperative Oncology Group PS of 0 to 2
6. Completion of IC to this trial

### 4-2 Exclusion criteria

Patients with either follow item have to be excluded from this trial.

1. Patients with necessity to heparinize during endoscopy
2. Patients with severe heart, lung and renal dysfunction
3. Patients with severe constipation
4. Debilitated patients who cannot undergo endoscopic or surgical resection
5. Inappropriate patients by each investigator's judgement

## 5. Study design

Multicenter, prospective comparative trial

## 6. Method for the study and enrollment

### 6.1. Standardization of diagnosis among endoscopists

All endoscopists attended the consensus meeting and were trained before the trial to standardize diagnosis shown in 7.2.2 among examiners, and this trial will start after achievement of good agreement among all participating examiners based on κ value.

## 6.2. Randomization

Enrolled patients were randomly assigned to A and B groups using a computer-aided system at the central center.

## 6.3. Enrollment

1. Each institution can start this trial after submitting a copy of each institution's IRB approval document to the central office.
2. The investigator of each institution starts IC and writes the patient's name in a screening list of each institution.
3. The manager of each institution number patients with IC using code of each institution.
4. The investigator faxes this code to the central office.
5. The central office faxes each institution the study group (A or B) that is randomly assigned, with trial registration number.
6. The investigator and assistant writes the results in the CRF.
7. The manager of each institution strictly keeps the screening list and CRF.
8. The investigator starts the trial as soon as possible after enrollment.

# 7. Method of the trial

## 7.1. Protocol for diagnosis

According to the following diagnostic protocol, the investigator diagnoses invasion depth after standard endoscopic observation.

### 7.1.1. A group: MC→EUS

1. The investigator diagnoses invasion depth by MC after spraying 0.05% crystal violet on the tumor and takes reliable pictures for the diagnosis.
2. The assistant measures and writes observation time of MC (min, sec) from starting spray to finishing observation.
3. The assistant writes investigator's diagnosis of invasion depth by MC in the CRF.
4. The investigator diagnoses invasion depth by EUS after suction of crystal violet and immersion of distilled water, and takes reliable pictures for the diagnosis.
5. The assistant measures and writes observation time of EUS (min, sec) from starting injection of distilled water to finishing observation.
6. The assistant writes investigator's diagnosis of invasion depth by EUS in the CRF.

### 7.1.2. B group: EUS→MC

1. The investigator diagnoses invasion depth by EUS after immersion of distilled water and takes reliable pictures for the diagnosis.
2. The assistant measures and writes observation time of EUS (min, sec) from starting injection of distilled water to finishing observation.
3. The assistant writes investigator's diagnosis of invasion depth by EUS in

the CRF.

4. The investigator diagnoses invasion depth by MC after suction of distilled water and spraying 0.05% crystal violet, and takes reliable pictures for the diagnosis.
5. The assistant measures and writes observation time of MC (min, sec) from starting spray to finishing observation.
6. The assistant writes investigator's diagnosis of invasion depth by MC in the CRF.

### 7.1.3. Tumor resection after examination

1. The investigator sends CRF to the central office within 7 days after examination.
2. Each tumor is appropriately resected with endoscopy or surgery based on the results of diagnosis and physical condition.

### 7.1.4. Submission of tissue samples

1. The investigator sends formalin fixed paraffin embedded (FFPE) sample slides of CRC tissues to the central office: 3 slides per each section.
2. (Sending address)
   Takaya Shimura, Masahide Ebi
   Department of Gastroenterology and Metabolism, Nagoya City University Graduate School of Medical Sciences, 1 Kawasumi, Mizuho-cho, Mizuho-ku, Nagoya 467-8601, Japan
3. Independent pathologist, who is blinded to all clinical data, pathologically diagnoses invasion depth, vascular infiltration and budding.

### 7.1.5. Storage of tissue samples

Tissue sample slides are stored in Department of Gastroenterology and Metabolism, Nagoya City University Graduate School of Medical Sciences

## 7.2. Diagnosis of invasion depth

### 7.2.1. Classification

Based on the difference of treatment strategy, diagnosis of invasion depth is categorized into 2 groups: 1. M/$SM_S$; 2. ≥$SM_D$.

### 7.2.2. Definition

① **MC**

**M/$SM_S$:** Type IIIs, $III_L$, IV pit pattern,
Type $V_I$ with low grade irregular pit pattern ($V_I$-L)

**≥$SM_D$ :** Type $V_I$ with high grade irregular pit pattern ($V_I$-H),
Type $V_N$ pit pattern (Non-structure)

② **EUS**

**M/SM$_S$**: A hypoechoic area limited to the 1st/2nd layers or with slight irregularity on the surface of the 3rd layer

**≥SM$_D$**: A hypoechoic area that clearly invades and penetrates into the 3rd layer

**8. The time course of the examination and observation**

| Observation items | Pre-IC | At the time of IC | Under colonoscopy | 30min after the trial |
|---|---|---|---|---|
| Patient background | ● | | | |
| IC for Colonoscopy | ● | | | |
| IC for the trial | | ● | | |
| Enrollment | | ● | | |
| Check for abdominal pain and bleeding | | ● | | ● |
| Oxygen saturation | | | ● | |
| Blood pressure | | | ● | ● |
| Diagnosis of invasion depth by MC and EUS | | | ● | |
| Observation time by MC and EUS | | | ● | |

**9. Prohibited combination drugs or treatments**

No set up

**10. Discontinuation of the trial**

1) The request to stop the trial from patients

2) Appearance of severe adverse events for continuing the trial

3) Other cases by the investigator's judgement

**11. Endpoint**

**11-1 Primary endpoint**

**Accuracy of invasion depth:**

Accuracy rate of invasion depth between prediagnosis of MC and EUS by comparison with the final pathological diagnosis (M/SM$_S$ or ≥SM$_D$)

**11-2 Secondary endpoint**

**Sensitivity and specificity for ≥SM$_D$:**

Sensitivity and specificity of prediagnosis by MC and EUS for the final pathological diagnosis of≥SM$_D$

**Observation time**: Time from starting MC or EUS to finishing observation

・**MC**：From spraying crystal violet to finish (min, sec)
・**EUS**：From injection of distilled water to finish (min, sec)

**Accuracy rate between MC in A group and EUS in B group**：
Accuracy rate of the primary diagnostic method in each group

**Accuracy rate in A and B group between MC and EUS**：
Accuracy rate in each group between MC and EUS

## 12. Adverse events and safety insurance for the trial

### 12-1 Definition of adverse events

All harmful events during the trial, regardless of the presence or absence of causual relationship

### 12-2 Assessment

・Assessment using the following NCI Common Terminology Criteria for Adverse Events v4.0 (CTCAE v4.0) as toxicity grading criteria

・Record in a patient's case record when adverse events are observed.

### 12-3 Assessment and report of severe adverse events

Definition of severe adverse events

① Fatality
② Life crisis
③ Need of hospitalization for treatment
④ Permanent or serious functional damage
⑤ Other serious medical event and reaction

・All adverse events within 30 minutes after the trial should be reported. However, events after 30 minute should be reported when relationship to the trial is suspicious.
・A managing investigator in each institution sends the detailed documentation of severe adverse events to the central office.
・A managing investigator in each institution has to report severe adverse events to the director of each hospital.

### 12-4 Expectable adverse events

The following adverse events may happen as well as standard colonoscopy: Perforation, Bleeding, Ileus, Thrombosis, Ischemic heart disease, Arrhythmia, Ischemic cerebrovascular disease, Abnormal body temperature, Abnormal blood pressure, Anaphylactic reaction

## 13. Criteria to stop the trial

### 13-1 Finish of the trial

The principal investigator will finish the trial after planned observation and examination.

### 13-2 Stop and suspension of the trial

The principal investigator has to report to each institution's managing investigator with a certain document when the trial is stopped or suspended by some issues of safety and efficacy.

## 14. Data publication

This clinical trial will be registered with University hospital Medical Information Network (UMIN).

The investigator will publish all or a part of results from this trial in the meeting and/or scientific medical journal.

## 15. Trial term

February, 2011 – December 31, 2013

## 16. Statistics

### 16-1 Main analysis and judgement

Data of accuracy, the primary endpoint, are analyzed using the $\chi^2$ test and values of $P < 0.05$ are defined significant.

### 16-2 Sample size

This trial verifies superiority of MC (experimental arm) to EUS (reference arm) for diagnostic accuracy of invasion depth.

It was estimated that MC would increase the accuracy for prediction of invasion depth of EUS from 70% to 90%. Sixty-two patients for each method are necessary to ensure a power of 80% for a two-sided 5% significance level test, and the planned sample size was 70 patients for each method, allowing for about a 10% dropout rate.

### 16-3 The final analysis

The final analysis calculates the diagnostic accuracy for invasion depth which is the primary endpoint.

## 17. Ethics

### 17-1 Protocol

All attending investigators have to follow the protocol.

### 17-2 Regulations (Protection of subjects)

Based on the ethical guidelines of the Declaration of Helsinki, the investigator considers human rights, safety and welfare of patients, respects for privacy and maintains secrecy.

Data of all patients are anonymously managed with blind registeration numbers

in the central office.

### 17-3 Informed consent

The investigator explains the content of this trial based under the IC documet, sign up to the IC document with the date and receive a sign of consent of patient’s own free will. The IC documents are kept in both patient and hospital.

### 17-4 IC document

1. An outline of this clinical trial
2. Aim of this clinical trial
3. Name of investigators in this clinical trial
4. Method and term of this clinical trial
5. Expectable advantage and disadvantage
6. Explanation about other diagnostic methods
7. Patients dose not receive any disadvantages if they do not attend this clinical trial.
8. Protection patient privacy
9. Compensation of health damage
10. Contact information about this trial (telephone number and address)
11. Cost relating to this clinical trial
12. Others (new information and possibility of stop)

The primary investigator amends the IC document when new important information are clarified, and it has to be approved by the IRB again.

### 17-5 Privacy

This trial preserves patient anonymity.

## 18. Compensation

This trial does not specially compensate for treatment costs when any incidents or accedints happen to patients during this trial.

## 19. Funding

### 19-1 Conflict of interests

There are no conflict of interests and no funding supports in this trial

### 19-2 Cost relating to this trial

Cost relating to this clinical trial owes to patients’ Medicare.
Cost to send slides of tumor samples owes to each institution.

## 20. Rivision of the protocol

We need the IRB approval again when the protocol will be amended.

## 21. Research organization

### 21-1 Principal investigator

Takaya Shimura
Department of Gastroenterology and Metabolism, Nagoya City University Graduate School of Medical Sciences, 1 Kawasumi, Mizuho-cho, Mizuho-ku, Nagoya 467-8601, Japan
TEL: +81-52-853-8211, FAX: +81-52-852-0952

**21-2 Research central office**

Department of Gastroenterology and Metabolism, Nagoya City University Graduate School of Medical Sciences, 1 Kawasumi, Mizuho-cho, Mizuho-ku, Nagoya 467-8601, Japan
TEL: +81-52-853-8211, FAX: +81-52-852-0952

**21-3 Drafting the protocol**

Takaya Shimura
Department of Gastroenterology and Metabolism, Nagoya City University Graduate School of Medical Sciences

**21-4 Pathologist**

Satoru Takahashi
Department of Experimental Pathology and Tumor Biology, Nagoya City University Graduate School of Medical Sciences

## 22. Research institutions and managing investigator

| | |
|---|---|
| Nagoya City University Hospital | Masahide Ebi, Takaya Shimura |
| Nagoya Daini Red Cross Hospital | Tomonori Yamada |
| Chukyo Hospital | Shozo Togawa |

## 23. References

1. Fujimori T, Kawamata H, Kashida H. Precancerous lesions of the colorectum. J Gastroenterol 2001; 36: 587-94.
2. Nascimbeni R, Burgart LJ, Nivatvongs S, Larson DR. Risk of lymph node metastasis in T1 carcinoma of the colon and rectum. Dis Colon Rectum 2002; 45: 200-6.
3. Kitajima K, Fujimori T, Fujii S, et al. Correlations between lymph node metastasis and depth of submucosal invasion in submucosal invasive colorectal carcinoma: a Japanese collaborative study. J Gastroenterol 2004; 39: 534-43.
4. Daichougan Kenkyukai. Daichougan chiryou guideline 2010. Kanehara shuppan 2010; Tokyo. (in Japanese)
5. Konishi K, Akita Y, Kaneko K, et al. Evaluation of endoscopic ultrasonography in colorectal villous lesions. Int J Colorectal Dis 2003; 18: 19-24.
6. Frascio F, Giacosa A. Role of endoscopy in staging colorectal cancer. Semin Surg Oncol 2001; 20: 82-5.
7. Kobayashi K, Kida M, Katsumata T, et al. Souki daichougan no shintatudoshindan oyobi chiryouhousentaku ni okeru chouonpanaisikyoushindan no

rinshouteki igi. Gastroenterol Endoscopy 2004; 46: 2132-40. (in Japenese)
8. Kato S, Fujii T, Koba I, et al. Assessment of colorectal lesions using magnifying colonoscopy and mucosal dye spraying: can significant lesions be distinguished? Endoscopy 2001; 33: 306-10.
9. Matsuda T, Fujii T, Saito Y, et al. Efficacy of the invasive/non-invasive pattern by magnifying chromoendoscopy to estimate the depth of invasion of early colorectal neoplasms. Am J Gastroenterol 2008; 103: 2700-6.
10. Bianco MA, Rotondano G, Marmo R, et al. Predictive value of magnification chromoendoscopy for diagnosing invasive neoplasia in nonpolypoid colorectal lesions and stratifying patients for endoscopic resection or surgery. Endoscopy 2006; 38: 470-6.
11. Matsumoto T, Hizawa K, Esaki M, et al. Comparison of EUS and magnifying colonoscopy for assessment of small colorectal cancers. Gastrointest Endosc 2002; 56: 354-60.
12. Hurlstone DP, Brown S, Cross SS, Shorthouse AJ, Sanders DS. High magnification chromoscopic colonoscopy or high frequency 20 MHz mini probe endoscopic ultrasound staging for early colorectal neoplasia: a comparative prospective analysis. Gut 2005; 54: 1585-9.
13. Fu KI, Kato S, Sano Y, et al. Staging of early colorectal cancers: magnifying colonoscopy versus endoscopic ultrasonography for estimation of depth of invasion. Dig Dis Sci 2008; 53: 1886-92.
